# Van der Woude syndrome and amniotic band sequence: A clue to a common genetic etiology? A case report

**DOI:** 10.1590/1678-4685-GMB-2024-0123

**Published:** 2025-02-17

**Authors:** Ana Luiza Bossolani-Martins, Joanna Goes Castro Meira, Gerson Shigeru Kobayashi, Adriana Barbosa-Gonçalves, Maria Rita Passos-Bueno, Agnes Cristina Fett-Conte

**Affiliations:** 1 Universidade Federal de Mato Grosso do Sul, Paranaíba, MS, Brazil.; 2 Universidade do Estado da Bahia, Salvador, BA, Brazil.; 3 Universidade de São Paulo, Instituto de Biociências, Centro de Estudos do Genoma Humano e Células-Tronco, São Paulo, SP, Brazil.; 4 Faculdade de Medicina de São José do Rio Preto, Hospital de Base, São José do Rio Preto, SP, Brazil.

**Keywords:** Cleft syndrome, autism spectrum disorder, interferon regulatory factor-6, missense variant, dysmorphic facial features

## Abstract

Rare heterozygous variants in *IRF6* (interferon regulatory factor-6) gene cause van der Woude syndrome 1 (VWS1) or Popliteal Pterygium syndrome, two forms of syndromic cleft lip/palate (CLP) that present with a variety of congenital malformations due to impairment ectodermal homeostasis. These malformations include, in addition to CLP, lip pits, pterygia, and intraoral and eyelid fibrous bands. Amniotic band sequence (ABS) is a rare condition of unknown genetic etiology that involves a range of congenital anomalies caused by the entanglement of fibrous bands, which disrupt fetal body parts. However, ABS co-occurs with CLP and other malformations that cannot be explained by this mechanism. Therefore, investigating the genetic relationship between ABS and CLP may provide clues regardind the genes involved in these conditions. Here, we report a case of a girl diagnosed with VWS1, autism, intellectual disability, and congenital right limb anomalies compatible with ABS. Molecular analysis revealed a novel, rare heterozygous missense variant in *IRF6* (NM_006147.3:c.970T>C) located in exon 7, inherited from her father. This variant results in the replacement of serine by proline at position 324 of the IRF6 protein with potentially deleterious effects. This report expands the mutational landscape of *IRF6* and provides further support for a possible link between the genetics of CLP and ABS.

## Introduction

Van der Woude syndrome 1 (VWS1; OMIM #119300) is a developmental disorder with an overall prevalence of 1:35,000 - 1:100,000 live births (Cervenka *et al*., 1967; Gorlin *et al*., 2001). It is a rare autosomal dominant inherited disorder mainly characterized by orofacial manifestations, such as fistulae (pits) on the paramedian portion of the vermillion border of the lower lip, with or without spittle excretion; cleft lip and/or cleft palate (CLP); hypodontia; abnormalities of limbs, skin, and nails; hearing deficits; congenital heart disease; cognitive deficits; and cerebral abnormalities (enlarged volumes of the anterior regions of the cerebrum) (Huang *et al*., 2007; Nopoulos *et al*., 2007b a). VWS1 presents high penetrance (80% to 90%) and variable expressivity, with familial recurrence observed in 61% of cases (van der Woude, 1954; Kantaputra *et al*., 2002; Rizos and Spyropoulos, 2004; Nopoulos *et al*., 2007b; Angiero *et al*., 2018). An allelic syndrome to VWS is Popliteal pterygium syndrome (PPS; OMIM #119500), which additionally includes popliteal pterygium, syndactyly, distinct toe/nail abnormality, syngnathia, genitourinary malformations, and risk of delayed language development, learning disabilities, and other mild cognitive problems (Wong & Hägg, 2004; Mujezinović, 2017). Both VWS and PPS may present with oral and eyelid fibrous bands (Bennun *et al*., 2018). 

Most of the VWS cases are caused by haploinsufficiency of the interferon regulatory factor-6 gene (*IRF6*), located on 1q32.2 (VWS1; OMIM #119300). *IRF6* belongs to a family of transcription factors that share a highly conserved N-terminal DNA-binding domain (DBD; exon 3-4) and a less-conserved C-terminal SMAD-interferon regulatory factor binding domain (SMIR/IAD; exon 7-9), both of which are mutational hot spots in VWS1. Missense, nonsense, frameshift, microdeletions and splicing variants in *IRF6* have been reported to cause VWS1 (de Lima *et al*., 2009; Little *et al*., 2009). *IRF6* coordinates epithelial proliferation/differentiation, and mutations in this gene are responsible for the alterations in ectoderm-derived tissues observed in VWS1 and PPS (Moretti *et al*., 2010; Thomason *et al*., 2010).

Amniotic band sequence (ABS; OMIM %217100) is a rare congenital disorder that affects between 0.19 and 8.1 per 10,000 births (López-Muñoz and Becerra-Solano, 2018). ABS refers to a spectrum of congenital anomalies associated with amniotic bands that entangle body parts, leading to tissue disruption. ABS typically presents with constriction rings and limb/digital amputations, with the addition of body wall, neural, spine or craniofacial defects in some cases (Seeds *et al*., 1982; Levy *et al*., 2007; López-Muñoz and Becerra-Solano, 2018; Singh and Gorla, 2022). To date, no underlying genetic mechanisms have been identified.

Here, we report a girl with VWS1, clinical features of ABS, autism spectrum disorder (ASD) and intellectual disability (ID), harboring a rare, novel missense *IRF6* variant. VWS1 and ABS are rare conditions that may share causative factors, and reporting new cases where they co-occur may provide grounds to elucidate etiological mechanisms.

## Subject and Methods

### Clinical evaluation

At the age of nine, the female patient was referred to the Genetics Clinic of the Medical School of São José do Rio Preto, São Paulo, Brazil due to severe autistic behavior. She was the second daughter of a non-consanguineous marriage, and her mother was 27 years old and her father was 26 years old. She was born at term (birth weight: 2.280 g, birth length: 47.5 cm) and there was no teratogenic exposure or any complications during pregnancy and delivery. 

At the first evaluation, at 9 years of age, she presented with short stature (3rd percentile), flat midface, narrow palpebral fissures, bilateral lower lip pits, submucous cleft palate, bifid uvula, hypodontia (missing right upper canine), congenital right limb anomalies suggestive of ABS (reduction and ring-like finger constrictions), ID and ASD. According to her parents, the developmental milestones were normal until about one year of age, after which they noticed developmental regression with onset and progression of autistic-like behaviors, such as deficit in social communication, repetitive behaviors (including hand flapping), absence of speech, self-injury, hyperactivity, irritability, mood disorder and tantrums. At the age of 3 years, she was diagnosed with ASD. Magnetic resonance imaging of the brain showed normal results. Her father presented only bilateral pits in the lower lip, and had normal neuropsychomotor development. Her sister had bilateral pits and sinuses in the lower lip, and learning disabilities. The patient completed the period of regular formal schooling (9 years) but with great difficulty. She did not agree to undergo a psychological assessment. They received the clinical diagnosis of VWS1. At the age of 17 years, a second examination of the patient additionally revealed motor impairment (march with hip swivel), cervical kyphosis and thoracolumbar scoliosis, and maintenance of important clinical and behavioral characteristics, such as short stature associated with ASD and severe cognitive impairment. 

**Figure 1 -:**
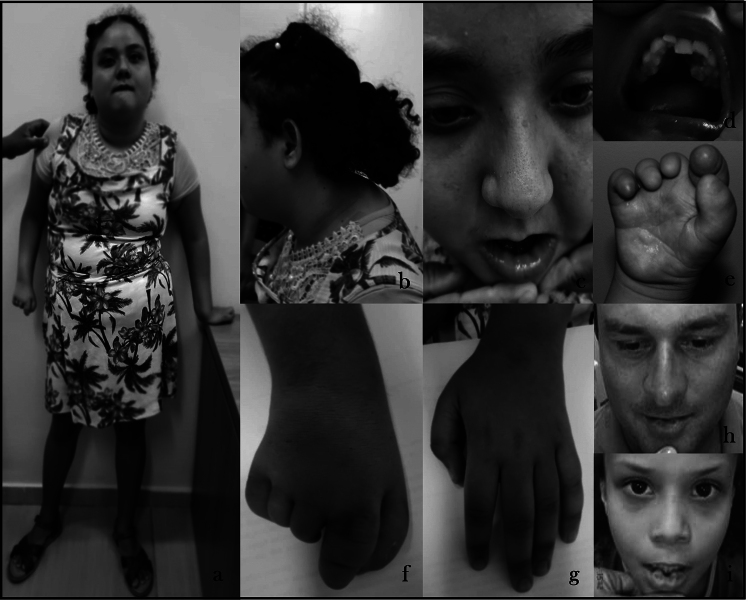
Patient presenting with short stature and kyphoscoliosis (a and b), lower lip pits (c), hypodontia (absence of the upper right canine tooth) (d), alteration of right hand with reduction and ring-like finger constrictions (e and f) and normal left hand (g). The father (h) and the sister (i) presenting with bilateral lower lip pits. Informed consent was obtained from the patient's legal guardian authorizing the publication of these images. Eyes were not blackened because they contain relevant clinical information.

Figure 1 shows the clinical characteristics of the patient at 17 years of age, as well as of her father and sister after surgical excision of the lower lip pits.

### Cytogenetic and molecular analysis

The patient’s peripheral blood karyotype was normal female (46, XX). The array Comparative Genomic Hybridization (Agilent SurePrint G3 Human CGH Microarrays 4×180K [180,000 oligonucleotide probes]) for screening of Copy Number Variations (CNVs) was applied (Supplementary Material Text S1). The analysis found no clinically significant CNVs. 

We performed sequencing analysis of the *IRF6* gene, including coding exons 3 to 8 and a portion of exon 9; the 5' untranslated region (5'UTR), which includes exons 1 and 2; a portion of the 3' untranslated region (3'UTR) comprising part of exon 9; and the sites of the exon-intron splice extending up to about 100bp on flanking intronic sequences. An extension of 143bp corresponding to the upstream region of *IRF6* was also sequenced (Supplementary Material Text S1). 

Nine Single Nucleotide Variants (SNVs) in *IRF6* have been identified in the proband*:* eight known variants and one variant not previously described in the literature, including ClinVar (Landrum *et al*., 2016), VarSome (Kopanos *et al*., 2019), gnomAD, and the Professional Human Gene Mutation Database (HGMD).

**Figure 2 -:**
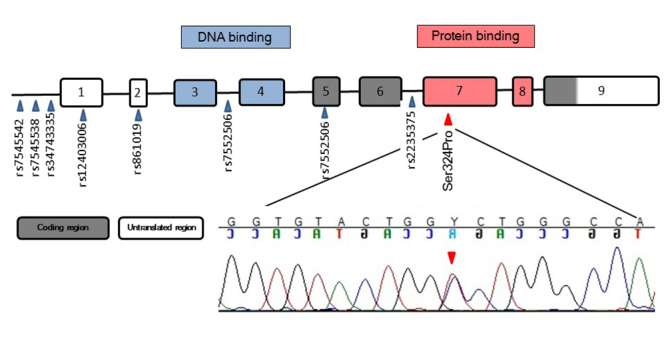
Representation of the structure of the *IRF6* gene showing all the variants found in the proband. The missense mutation in exon 7 occurs at the binding site of IRF6 protein, where serine is replaced by (red arrow). Chromatogram of exon 7 in the *IRF6* gene, illustrating the novel heterozygous missense mutation (T>C) characterized as NM_006147.3: c.970T> C (red arrow).

This novel variant is a heterozygous missense mutation, NM_006147:c.970T>C, (DNAg1274T>C, NC_000001.10, g209963930A>G). This SNV is located in exon 7 within the IRF6 SMIR/IAD domain (Figure 2) and it leads to the replacement of serine by proline at position 324 (NP_006138.1; p.Ser324Pro).

All variants were classified on VarSome (Kopanos *et al*., 2019) according to the American College of Medical Genetics and Genomics (ACMG) standards and guidelines for the interpretation of variants (Richards *et al*., 2015), and based on the following criteria: PM1 (location in mutational hot spot or well-established functional domain), PM2 (frequency in control databases, e.g. 1000 Genomes, ExAC, ESP), PP3 (computational evidence, e.g. conservation, splicing impact, etc), PP1 (co-segregation with disease in family members in a disease-associated gene), and PP4 (disease-specific patient’s phenotype or family history). The novel variant NM_006147:c.970T>C was classified as likely pathogenic, and was not present in any control database, including the Brazilian population (ABraOM) (Naslavsky *et al*., 2022). The other eight variants were classified as benign or likely benign.

Sequencing of the proband’s relatives revealed that her father and sister, but not her mother, carried the same c.970T>C variant, compatible with their VWS1 clinical presentation. The mother, father and sister shared nearly all of the benign/likely benign variants detected in the proband (Table 1).

**Table 1 -:** SNVs found in the *IRF6* gene sequencing of the proband.

Single Nucleotide Variant^1^	Gene region	Genomic location^2^ (GRCh37/hg19)	mRNA location^2^	ACMG prediction	ClinVar status	Publications associated with oral clefts
rs7545542	upstream	g.209979635C>T	c.-304-115G>A	Likely Benign	Not described	Not described
rs7545538	upstream	g.209979613C>G	c.-304-93G>C	Benign	Not described	Mijiti *et al*., 2015
rs34743335	upstream	g.209979529A>T	c.-304-9T>A	Likely Benign	Benign	Not described
rs12403006	5’UTR (Exon 1)	g.209979518T>A	c.-302A>T	Benign	Benign	Not described
rs861019	5’UTR (Exon 2)	g.209975386A>G	c.-73T>C	Benign	Benign	Vieira *et al*., 2007; Pegelow *et al*., 2008
rs7552506	Intron 3	g.209969902G>C	c.175-5 C>G	Benign	Benign	Zechi-Ceide *et al*., 2007; Pegelow *et al*., 2008
rs2013162	Exon 5	g.209968684C>A	c.459G>T p.Ser153=	Benign	Benign	Scapoli *et al*., 2005; Park *et al*., 2007; Marazita *et al*., 2009; Lu *et al*., 2013; Wattanawong *et al*., 2016; Xu *et al*., 2016; Soleymani *et al*., 2022
rs2235375	Intron 6	g.209965587G>C	c.667+27C>G	Benign	Benign	Scapoli *et al*., 2005; Huang *et al*., 2009; Gurramkonda *et al*., 2018; Suazo *et al*., 2020; Soleymani *et al*., 2022
Novel variant	Exon 7	g209963930A >G	c.970T>C p.(Ser324Pro)	Likely Pathogenic	Not described	Not described

^1^ All variants except for c.970T>C (novel variant) were shared by family members. rs7545542, rs7545538, rs34743335, rs12403006 and rs8610192 were shared by the father and sister, while the mother was not evaluated. rs7552506, rs2013162 and rs2235375 were shared by the mother, father and sister.

^2^ Accession numbers of the reference sequences of genomic DNA regarding the chromosome, complementary DNA and protein were NC_000001.10, NM_006147.3 and NP_006138.1, respectively (http://www.ncbi.nlm.nih.gov/RefSeq).

We obtained the approval of the Human Ethics Committee of the School of Medicine of Sao Jose do Rio Preto (FAMERP), State of Sao Paulo, Protocol nº 3306/2010. Informed consent was obtained from the patient’s father (legal guardian) for the publication of this case report and its accompanying images.

## Discussion

At least 200 different variants in the *IRF6* gene have been described, most of which are missense or protein truncation mutations (nonsense and frameshift). Overall, 78% of the VWS1 pathogenic mutations are located in exons 3, 4 and 7 (Kondo *et al*., 2002; de Lima *et al*., 2009). Here, we report a novel and rare heterozygous missense variant in *IRF6* exon 7, c.970 T>C:p.(Ser324Pro), classified as likely pathogenic.

Previous work indicates that children with isolated orofacial clefts may have increased risk for neurodevelopmental disorders, including ASD and ID (Tillman *et al*., 2018), while lower cognitive function and structural brain alterations have been observed both in individuals with isolated CLP and VWS1 (Nopoulos *et al*., 2002; Nopoulos *et al*., 2007a, b). Here, the patient has ASD and ID that could not be explained by CNVs, but we cannot rule out the contribution of other genetic and/or environmental factors. Therefore, whether haploinsufficiency of *IRF6* is a risk factor for these neuropsychiatric phenotypes remains to be confirmed.

To date, this is the second report of an ABS-like presentation in a VWS patient. Kuster and Lambrecht (1988) reported a girl with VWS and ABS, whose hands showed a similar presentation to that of our patient's right hand. Considering the overall birth prevalence of VWS and ABS, the probability of co-occurrence of these syndromes would roughly fall between 1:40,000,000 and 1:5,000,000,000, which would arguably be a considerably rare, or even unlikely event if independent etiologies are regarded.

Although “classical” ABS is considered to originate via disruption of fetal tissue by amniotic bands, some congenital defects present in part of the ABS cases do not seem to follow this paradigm. These include malformations such as CLP and congenital heart defects, which are developmental phenotypes (Robin *et al*., 2005). Notably, this observation has led some authors to suggest that a possible etiological overlap may be a clue to the genetic etiology of ABS, especially in the case of syndromic CLP forms associated with oral and facial fibrous bands. These include VWS/PPS and Hay-Wells syndrome, caused by mutations in *TP63* (tumor protein p63) (Robin *et al*., 2005; Cignini *et al*., 2012). Interestingly, while it has been hypothesized that ABS originates from early ectodermal defects (Hunter *et al*., 2011), *IRF6* and *TP63* are known to interact in a regulatory loop responsible for ectodermal homeostasis that, when disturbed, may result in either IRF6- or *TP63*-associated phenotypes (Moretti *et al*., 2010). Therefore, one may speculate that disturbances in this regulatory mechanism may also play a role in at least part of the ABS cases.

In summary, we report a novel pathogenic variant in *IRF6*, expanding the mutational landscape of VWS1 and PPS. Importantly, disturbances in *IRF6* functions during embryonic development may be an underlying cause of both VWS1 and ABS, and exploring this possibility may advance understanding of their pathogenic mechanisms.

## Supplementary material

The following online material is available for this article:

Text S1 -
*IRF6* sequencing and screening for CNVs.
