# Supplementary material for: Van der Woude syndrome and amniotic band sequence: A clue to a common genetic etiology? A case report
Source: Genet Mol Biol. 2025 Feb 17;48(1):e20240123. doi: 10.1590/1678-4685-GMB-2024-0123 (PMC11895807; doi:10.1590/1678-4685-GMB-2024-0123)
Supplement: Text S1 - [file 1415-4757-GMB-48-1-e20240123-s1.pdf]

Genetics and Molecular Biology
https://doi.org/10.1590/1678-4685-GMB-2024-0123

# Supplementary Material to "Van der Woude syndrome and amniotic band sequence: A clue to a common genetic etiology? A case report"

Text S1 - *IRF6* sequencing and screening for CNVs

The patient was identified from a genetic study of individuals with the diagnosis of ASD. A clinical evaluation of the patient was performed, and subsequently, her blood samples underwent cytogenetic analysis, sequencing of the *IRF6* gene for mutation screening and aCGH.

The Sanger sequencing of *IRF6* (1q32.3-q41) included conducting nine PCRs, product purification by Exo-Sap ®(Affymetrix USB®), sequencing reaction and purification of the product by Sephadex® (GE®). The samples were submitted to capillary electrophoresis in an ABI 3730 DNA Analyser automatic sequencer (Applied Biosystems®), and the results were analyzed with the Sequencher® program (version 5.1; Gene Codes Corporation). The primers used contained the M13 tail and its sequence and programming for the thermocycler can be obtained upon request.

For screening of CNVs, the aCGH platform (Agilent SurePrint G3 Human CGH Microarrays 4×180K [180,000 oligonucleotides probes]) was applied. The procedures for DNA digestion, labeling with Cy3 and Cy5-dCTPs by random priming followed by purification, hybridization and washing were performed according to the manufacturer's protocol (Agilent Technologies®). Scanned images of the arrays were processed using Feature Extraction software, v10.5 (Agilent Technologies, Santa Clara, CA), and the analysis was carried out using Agilent Genomic Workbench Lite Edition v6.5 (Agilent Technologies, Santa Clara, CA), using the ADM-2 statistical algorithm and the threshold 7.0.
